# Enzymatic control of anhydrobiosis-related accumulation of trehalose in the sleeping chironomid, *Polypedilum vanderplanki*

**DOI:** 10.1111/j.1742-4658.2010.07811.x

**Published:** 2010-10

**Authors:** Kanako Mitsumasu, Yasushi Kanamori, Mika Fujita, Ken-ichi Iwata, Daisuke Tanaka, Shingo Kikuta, Masahiko Watanabe, Richard Cornette, Takashi Okuda, Takahiro Kikawada

**Affiliations:** 1 Anhydrobiosis Research Unit, National Institute of Agrobiological SciencesTsukuba, Ibaraki, Japan; 2 Department of Integrated Biosciences, Graduate School of Frontier Sciences, The University of TokyoJapan

**Keywords:** anhydrobiosis, trehalase, trehalose, trehalose-6-phosphate phosphatase, trehalose-6-phosphate synthase

## Abstract

Larvae of an anhydrobiotic insect, *Polypedilum vanderplanki*, accumulate very large amounts of trehalose as a compatible solute on desiccation, but the molecular mechanisms underlying this accumulation are unclear. We therefore isolated the genes coding for trehalose metabolism enzymes, i.e. trehalose-6-phosphate synthase (TPS) and trehalose-6-phosphate phosphatase (TPP) for the synthesis step, and trehalase (TREH) for the degradation step. Although computational prediction indicated that the alternative splicing variants (*PvTpsα/β*) obtained encoded probable functional motifs consisting of a typical consensus domain of TPS and a conserved sequence of TPP, *PvTpsα* did not exert activity as TPP, but only as TPS. Instead, a distinct gene (*PvTpp*) obtained expressed TPP activity. Previous reports have suggested that insect TPS is, exceptionally, a bifunctional enzyme governing both TPS and TPP. In this article, we propose that TPS and TPP activities in insects can be attributed to discrete genes. The translated product of the TREH ortholog (*PvTreh*) certainly degraded trehalose to glucose. Trehalose was synthesized abundantly, consistent with increased activities of TPS and TPP and suppressed TREH activity. These results show that trehalose accumulation observed during anhydrobiosis induction in desiccating larvae can be attributed to the activation of the trehalose synthetic pathway and to the depression of trehalose hydrolysis.

## Introduction

The sleeping chironomid, *Polypedilum vanderplanki*, can withstand drought stress by the induction of an ametabolic state termed ‘cryptobiosis’ or ‘anhydrobiosis’ [1,2]. Many anhydrobiotic organisms, including bacteria, fungi, plants and invertebrates, are known to accumulate a nonreducing sugar, such as trehalose or sucrose, at high concentrations prior to or on desiccation [3,4], although several tardigrades, including *Milnesium tardigradum*, and bdelloid rotifers, including *Philodina roseola* and *Adineta vaga*, can enter anhydrobiosis without trehalose or trehalose accumulation [5,6]. Trehalose, the focus of this paper, is thought to effectively protect organisms from severe desiccation stress owing to its ability for water replacement and vitrification [3,4,7]. In *P. vanderplanki*, as larvae are undergoing desiccation, a large amount of trehalose is produced in the fat body cells [8] and redistributed to other cells and tissues through a facilitated trehalose transporter, TRET1 [9]. The transported trehalose has been shown to vitrify in the completely desiccated insects [7]. Thus, the mechanisms underlying the diffusion of accumulated trehalose over the entire insect body, and the protective effect of trehalose on cell components, have been established. Nevertheless, the molecular mechanisms involved in trehalose accumulation in *P. vanderplanki* remain obscure.

**Fig. 1:**
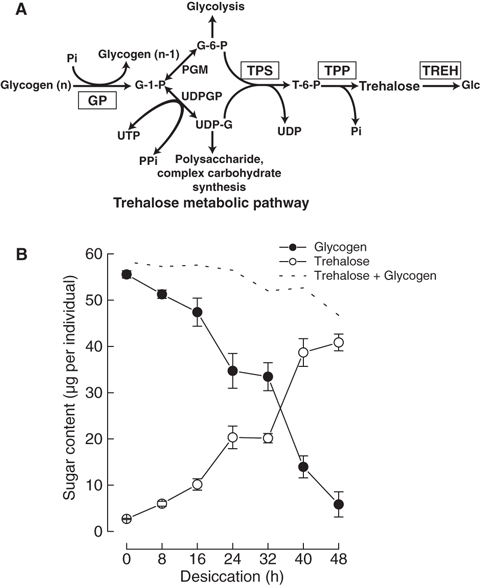
Schematic representation of the trehalose metabolic pathway (A) and changes in glycogen and trehalose content in *P. vanderplanki* larvae during desiccation treatment (B). Filled circles and open circles represent glycogen and trehalose content, respectively; the broken line represents the amount of total carbohydrate. G-1-P, glucose-1-phosphate; G-6-P, glucose-6-phosphate; Glc, glucose; PGM, phosphoglucomutase; UDPGP, UDP-glucose pyrophosphorylase; Pi, inorganic phosphate; PPi, pyrophosphate; T-6-P, trehalose-6-phosphate.

In addition to its role as an anhydroprotectant, trehalose is generally known as a carbon and energy source for bacteria and yeast [10]. In bacteria and yeast, trehalose is synthesized from glucose-6-phosphate and UDP-glucose, catalyzed by trehalose-6-phosphate synthase (TPS; EC 2.4.1.15) and trehalose-6-phosphate phosphatase (TPP; EC 3.1.3.12), and the relevant genes have been cloned and well characterized (Fig. 1A). This synthetic pathway is considered to be conserved in a wide range of taxa, including unicellular and multicellular organisms, because these genes have been found in algae, fungi, plants and invertebrates [11].

In numerous insect species, trehalose is the main hemolymph sugar, although many exceptions, including dipteran, hymenopteran and lepidopteran species, have been reported to contain both trehalose and glucose and even to completely lack trehalose, depending on the physiological conditions [12,13]. Trehalose is synthesized predominantly in the fat body, and then released into the hemolymph. After uptake by trehalose-utilizing cells and tissues, trehalose is hydrolyzed to glucose by trehalase (TREH; EC 3.2.1.28). To date, TREH has been studied extensively in many insect species because of its role as the enzyme responsible for the rate-limiting step in trehalose catabolism in eukaryotes [12]. In *Bombyx mori*, *Tenebrio molitor*, *Pimpla hypochondriaca*, *Apis mellifera*, *Spodoptera exsigua* and *Omphisa fuscidentalis*, TREH genes have been cloned and demonstrated to be implicated in certain physiological events [12,14–18]. Several biochemical studies on insect TPS and TPP have been reported [12], but these are markedly less complete relative to those on TREH. *Tps* genes have been reported in many invertebrate species, including a model nematode, *Caenorhabditis elegans*, an anhydrobiotic nematode, *Aphelenchus avenae*, a crustacean, *Callinectes sapidus*, and insects, *Drosophila melanogaster*, *Helicoverpa armigera* and *Spodoptera exigua* [19–23]. Furthermore, insect genome projects have shown that *Tps* gene sequences are found in *Apis mellifera*, *Triboliumcastaneum*, *Locusta migratoria*, *Anopheles gambiae* and *Culex pipiens.* Among the insect genes, *Drosophila tps1* (*dtps1*) and *Helicoverpa Tps* (*Har-Tps*) are expressed heterologously, and TPS activity has been confirmed in the resultant proteins [21,22]. Furthermore, the effects of overexpression of *dtps1* on trehalose levels in relation to anoxia tolerance [21], and the involvement of *Har-Tps* in diapause induction [22], have been reported. No information on the insect *Tpp* gene has been obtained, but, instead, it has been suggested that DTPS1 and Har-TPS may act not only as TPS, but also as TPP [21–23]. The basis for this suggestion is that TPSs comprise both the Glyco_transf_20 (GT-20) motif responsible for trehalose-6-phosphate synthesis, and the trehalose_PPase (TrePP) motif, according to motif analysis on the Pfam (protein family) database (http://pfam.sanger.ac.uk/). However, on balance, the regulation of trehalose metabolism in insects has not been studied comprehensively.

Thus, the elucidation of how enzymes control the rapid accumulation of trehalose in response to desiccation stress should provide important information for understanding the molecular mechanism of anhydrobiosis induction in *P. vanderplanki* as well as fundamental insect physiology. In this study, we identified the genes involved in trehalose metabolism and analyzed their expression and the functions of the gene products.

## Results

### Changes in trehalose and glycogen contents in *P. vanderplanki* during desiccation

In insects, glycogen is the major substrate for trehalose synthesis [12,13,24]. During desiccation in *P. vanderplanki*, changes in trehalose and glycogen contents were correlated, i.e. the conversion of glycogen into trehalose (Fig. 1B). As the sum of trehalose and glycogen was fairly constant, the fluctuations in trehalose and glycogen contents during desiccation indicate that trehalose is likely to be synthesized from glucose-6-phosphate and UDP-glucose originating from the glycogen stored in fat body cells.

### Changes in the activities of trehalose metabolism enzymes in *P. vanderplanki* during desiccation

**Fig. 2:**
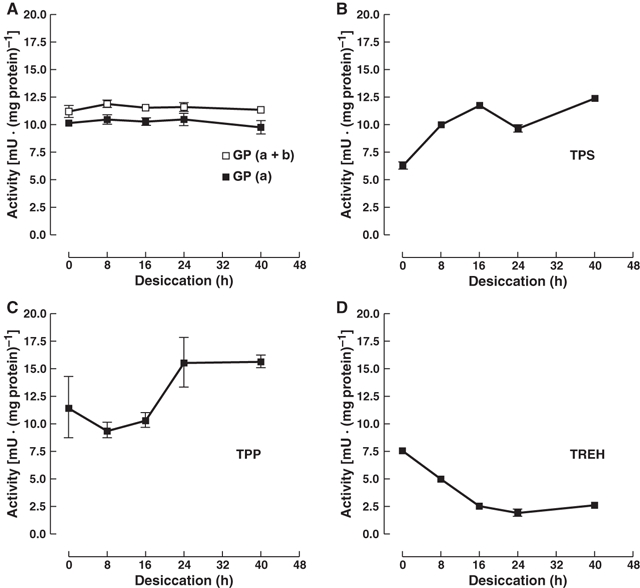
Changes in the activities of the enzymes involved in trehalose metabolism during desiccation. Using total protein extracted from the larvae sampled at various times of desiccation treatment, enzyme activities of GP (A), TPS (B), TPP (C) and TREH (D) were determined. In the GP assay, filled symbols represent the activity of the active form a, and open symbols represent the total activity including the inactive form b.

The activities of the enzymes involved in trehalose metabolism were investigated during the desiccation of *P. vanderplanki*. As desiccation progressed, the activities of TPS and TPP were enhanced prior to and parallel with trehalose accumulation, respectively, whereas TREH activity decreased (Fig. 2B–D). Glycogen phosphorylase (GP) activity is generally controlled not only by gene expression, but also by reversible phosphorylation. Thus, GPb (inactive form) is reversibly converted into GPa (active form) by phosphorylation. In the results of GP assays, the GPa activity and total activity originating from both forms of GP protein were constant throughout the desiccation process (Fig. 2A). These results indicate that changes in the activity of TPS, TPP and TREH, rather than GP, are responsible for the accumulation of trehalose originating from glycogen.

### Cloning of *PvTpsα/β*, *PvTpp* and *PvTreh* cDNA

To elucidate the molecular mechanisms of the enhancement of the trehalose biosynthetic activity during desiccation in *P. vanderplanki*, we cloned the genes for TPS, TPP and TREH.

Full-length cDNAs of *PvTps* and *PvTreh* were isolated by RT-PCR and/or 5′- and 3′-RACE. For the isolation of cDNAs, degenerated primer sets were designed on the basis of the nucleotide sequences of *Tps* and *Treh* cDNAs that have been reported previously in many organisms [12,25–32]. After cDNA fragments corresponding to each gene had been obtained, 5′- and 3′-RACE were performed. Information on the nucleotide sequence of *PvTpp* was obtained by screening in an expressed sequence tag (EST) database constructed with sequences of cDNAs prepared from desiccating larvae [33], and the full-length cDNA was determined by 5′-RACE.

**Fig. 3:**
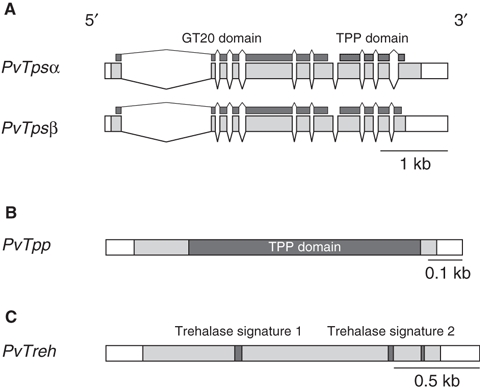
Schematic representation of desiccation-inducible genes isolated from *P. vanderplanki*. (A) Genomic structures of *PvTpsα* and *PvTpsβ*. Exons are indicated by boxes (shaded boxes corresponding to ORF) and introns by straight lines. Filled bars indicate representative motifs encoded in the genes. (B, C) Diagrams of cDNAs of *PvTpp* and *PvTreh*, respectively. Shaded regions indicate ORF. Filled boxes represent consensus motifs encoded in the nucleotide sequence. Scale bars are displayed at the bottom right of each diagram.

As a result of 3′-RACE on *PvTps*, we isolated two distinct mRNAs, named *PvTpsα* and *PvTpsβ*, that were different at each 3′-end of the nucleotide sequence. *PvTpsα* cDNA consisted of 3026 bp (Fig. 3A). Because nucleotides (nt) 69–71 represent a stop codon (TAA), the downstream nt 90–92 were regarded as the initiation codon (ATG). nt 2628–2630 also represented a stop codon (TGA), thus suggesting a 2538-bp ORF (846 amino acids with a molecular mass of 95 300). *PvTpsβ* cDNA consisted of 3094 bp; 68 nucleotides were inserted between nt 2291 and 2292 of *PvTpsα*. Because a frame shift occurred by insertion, the ORF in *PvTpsβ* was shortened to 2373 bp, encoding 791 amino acids with a calculated molecular mass of 89 500 (Fig. 3A). The genomic DNA sequence of the *PvTps* gene confirmed that *PvTpsα* and *PvTpsβ* were generated by alternative splicing (Fig. 3A). In the same manner, cDNAs of *PvTpp* and *PvTreh* were defined to consist of 1044 bp, including an 882-bp ORF (294 amino acids with a molecular mass of 33 400), and 2177 bp, including a 1734-bp ORF (578 amino acids with a molecular mass of 66 400), respectively (Fig. 3B, C).

The deduced amino acid sequences of PvTPSα/β, PvTPP and PvTREH were subjected to Pfam search. PvTPSα and PvTPSβ have both the GT-20 and TrePP motifs, whereas PvTPP has the TrePP motif only (Fig. 3A, B). The GT-20 motif, belonging to the glycosyl transferase family 20, is found in every TPS and several TPP proteins, and the TrePP motif is found in several TPSs and every TPP protein [32]. In PvTREH, we found TREH signature 1, TREH signature 2 and a glycine-rich region, which are the consensus sequences of the TREH protein (Fig. 3C). Thus, *PvTpsα/β*, *PvTpp* and *PvTreh* seemed to encode TPS, TPP and TREH, respectively, of *P. vanderplanki*.

### Functional analysis of *PvTpsα/β*, *PvTpp* and *PvTreh*

**Fig. 4:**
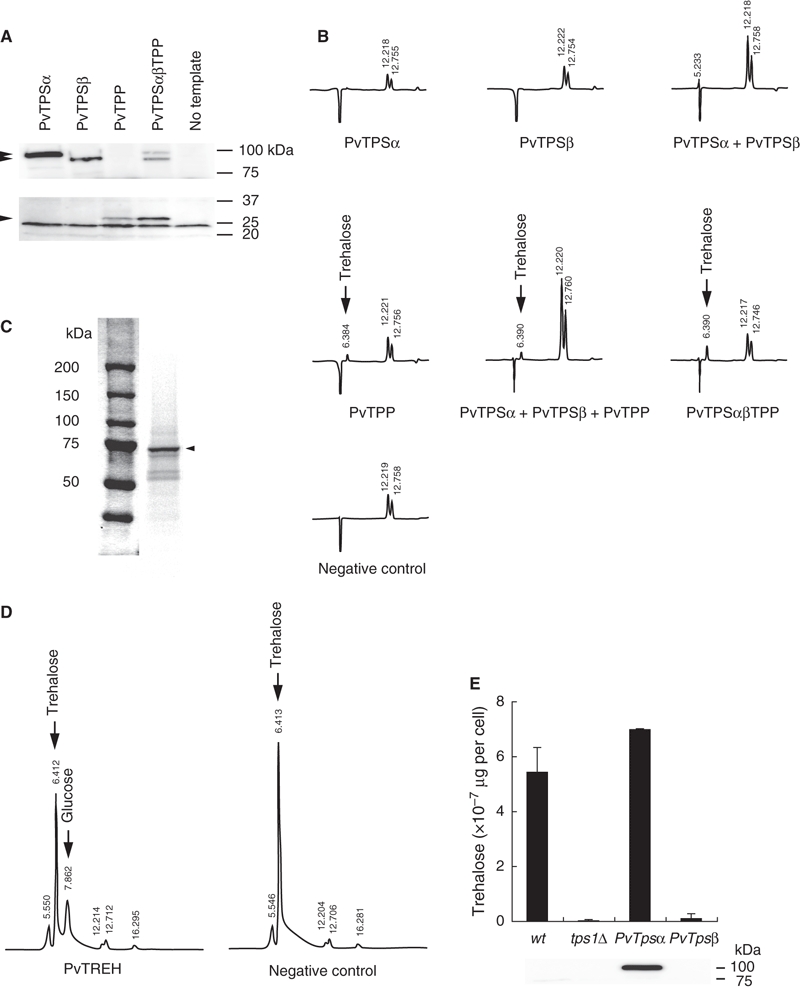
Functional analyses of PvTPSα, PvTPSβ, PvTPP and PvTREH proteins. (A, C) Confirmation of protein production by *in vitro* transcription and translation (A: PvTPSα, PvTPSβ and PvTPP; C: PvTREH). Aliquots of non-labeled or [^35^S]-labeled proteins were analyzed by SDS/PAGE and western blotting (A) or autoradiography (C). (B, D) HPLC analyses of the resultant products from enzymatic assays for TPP (B) and TREH (D). Arrowhead indicates the position of the target protein. Arrows represent the elution positions of trehalose and glucose. (E) Trehalose estimation in yeast transformants. Top: the ability to produce trehalose was evaluated in each yeast strain transformed with *PvTpsα/β*-containing vector. Bottom: western blot analysis of PvTPSα/β expression. Total protein was extracted from the aliquot of the culture used for trehalose measurement and subjected to SDS/PAGE and western blotting with anti-PvTPS IgG.

To corroborate whether these genes encode functional proteins, recombinant proteins were prepared using an *in vitro* transcription and translation system (TnT, Promega, Madison, WI). First, we checked that protein synthesis was successful via SDS/PAGE and western blot analysis (Fig. 4A). The expression of PvTPP protein was very faint. The coexistence of both *PvTpsα* and *PvTpsβ* cDNAs with *PvTpp* cDNA in the TnT reaction mixture was successful for the expression of these proteins, although the expression levels were slightly lower. In the TPS assay, PvTPSα and PvTPSβ showed no activity; trehalose-6-phosphate was not produced from glucose-6-phosphate and UDP-glucose (data not shown). TPS activity was also not detected when PvTPSβ and PvTPP were present with PvTPSα. In the TPP assay with PvTPP only, or mixed with PvTPSα and PvTPSβ, catalyzed dephosphorylation of trehalose-6-phosphate into trehalose occurred (Fig. 4B). As neither PvTPSα nor PvTPSβ (or both) was able to dephosphorylate trehalose-6-phosphate, we conclude that PvTPP is responsible for dephosphorylation. The incubation of PvTREH with trehalose resulted in the production of glucose, indicating that PvTREH functions as TREH by hydrolysis of the α-1,1-glycosidic bond in trehalose (Fig. 4C, D).

TPS activity was not detected in the recombinant PvTPSα or PvTPSβ*in vitro*. Genetic techniques using yeast deletion mutants are also a powerful tool for the functional analysis of TPS [34–36]. In order to confirm the function of PvTPSα and PvTPSβ, we employed yeast *tps1* deletion mutants. The yeast deletion mutant of TPS1 (*tps1Δ*), lacking the *TPS1* gene corresponding to TPS, was transformed with the *PvTpsα* or *PvTpsβ* expression vector. These transformants were examined for their ability to synthesize trehalose. The *tps1Δ*+ *PvTpsα* strain, but not the *tps1Δ*+ *PvTpsβ* strain, accumulated trehalose comparably to the wild-type (Fig. 4E). We checked the expression of the PvTPSα and PvTPSβ proteins in each transformant, and found that PvTPSα was successfully expressed, but that PvTPSβ was not (Fig. 4E). From these results, the catalytic activity of the PvTPSα protein was demonstrated, although the function of PvTPSβ as an enzyme was not shown.

### Complementation of the yeast *tps1* or *tps2* deletion mutant phenotype by the corresponding *PvTpsα* or *PvTpp* gene

**Fig. 5:**
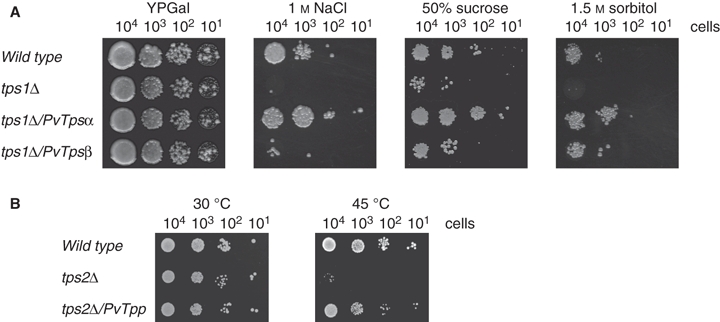
Complementation assay using yeast deletion mutants. (A) Complementation of *S. cerevisiae tps1* deletion mutant by *PvTpsα/β*. Yeast cells were grown on a plate containing YP medium with galactose (YPGal) under hyperosmotic conditions (1 m NaCl, 50% sucrose and 1.5 m sorbitol). (B) Complementation of *S. cerevisiae tps2* deletion by *PvTpp*. Yeast cells were plated on SD agar medium containing galactose and lacking uracil and methionine. To confirm whether the transformants rescued thermosensitivity, yeasts were incubated at 45 °C for 5 h and then grown at 30 °C. Representative results of three independent experiments are shown.

The yeast deletion mutant *tps1Δ* has been reported to be osmosensitive [34–36]. In the *tps2Δ* strain, the yeast deletion mutant lacking the *TPS2* gene corresponding to TPP, thermosensitivity to high temperature was reported [37,38]. Thus, we examined whether *PvTpsα/β* in *tps1Δ* and *PvTpp* in *tps2Δ* rescued the deletion mutants from osmosensitivity and thermosensitivity, respectively (Fig. 5). The *tps1Δ*+ *PvTpsα* strain grew at the same level as the wild-type on hypertonic medium containing 1 m NaCl, 50% sucrose or 1.5 m sorbitol (Fig. 5A). However, the *tps1Δ*+ *PvTpsβ* strain showed little improvement in growth rate compared with the *tps1Δ* strain on 1 m NaCl and 50% sucrose plates (Fig. 5A); these results are consistent with the absence of PvTPSβ expression (Fig. 4E). Nevertheless, *tps1Δ*+ *PvTpsβ* on 1.5 m sorbitol plates showed slightly lower growth than the *tps1Δ*+ *PvTpsα* strain (Fig. 5A). At present, we have no adequate explanation for this modest rescue; it may be caused by a kind of side-effect of transformation or the presence of trace amounts of the PvTPSβ protein.

Thermosensitivity in the *tps2Δ*+ *PvTpp* strain was rescued to almost the same level as the wild-type (Fig. 5B). These results clearly demonstrate that *PvTpsα* and *PvTpp* function genetically as *Tps* and *Tpp*, respectively.

### Expression profiles of *PvTpsα/β*, *PvTpp* and *PvTreh* mRNAs and proteins

**Fig. 6:**
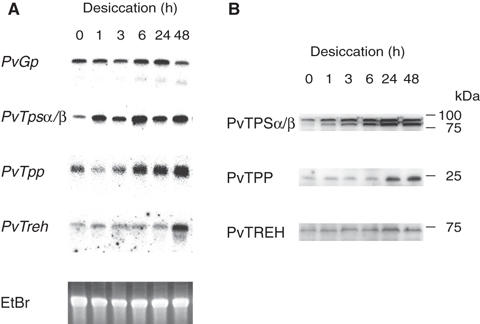
Expression profiles of mRNAs and proteins of the genes involved in trehalose metabolism during desiccation. Total RNA and protein were prepared from larvae treated under desiccation conditions, and analyzed by northern blotting (A) and western blotting (B).

As shown in Fig. 1B, in *P. vanderplanki*, trehalose is likely to be synthesized from glycogen en route to anhydrobiosis. In eukaryotes, the metabolic pathway from glycogen to trehalose is highly conserved (Fig. 1A). Hence, in order to elucidate the molecular mechanisms underlying the regulation of the enzymes involved in trehalose metabolism on desiccation, we first investigated the expression profiles of *PvTpsα/β*, *PvTpp* and *PvTreh* mRNAs (Fig. 6A). The accumulation of *PvTpsα/β* and *PvTpp* mRNAs was induced within 1 h and 3 h, respectively, during desiccation treatment. For *PvTreh*, the induction of mRNA accumulation was delayed by 48 h after the beginning of desiccation treatment compared with the other two genes. Real-time PCR analyses of these mRNAs confirmed the results (data not shown). However, the amount of *PvGp* mRNAs remained constant during treatment, which is consistent with the constancy of GP activity on desiccation (Fig. 2A). Western blot analyses revealed that the proteins of PvTPSα/β, PvTPP and PvTREH were also accumulated, as were the corresponding mRNAs (Fig. 6B).

## Discussion

In this study, we have isolated and characterized three desiccation-inducible genes, *PvTpsα/β*, *PvTpp* and *PvTreh*, encoding the enzymes involved in trehalose metabolism in *P. vanderplanki* (Fig. 3). In addition to *P. vanderplanki*, many anhydrobiotes, such as *A. avenae*, and *Artemia* cysts accumulate trehalose as they undergo desiccation. In these organisms, trehalose accumulation correlates significantly with anhydrobiosis induction [3,4,39]. In contrast, several rotifers and tardigrades enter anhydrobiosis without trehalose accumulation, but possess other anhydroprotectants, such as late embryogenesis abundant proteins [4,6]. The induction of trehalose synthesis is necessary for *P. vanderplanki* to achieve anhydrobiosis. The larvae, if rapidly dehydrated, cannot enter anhydrobiosis because of an insufficient amount of trehalose [40,41]. Furthermore, it has been hypothesized that trehalose is replaced with water or can vitrify to exert its protective function against dehydration [3,4,7]. Indeed, trehalose is produced in fat body cells in desiccating *P. vanderplanki* larvae [8], redistributed to other cells and tissues through a facilitated trehalose transporter, TRET1 [9], and vitrified in completely desiccated insects [7]. Thus, the successful induction of anhydrobiosis in *P. vanderplanki* must occur via a sequence of events: expression of trehalose metabolism-related genes, *de novo* synthesis and accumulation of trehalose, redistribution and vitrification.

*PvTpsα* rescued the growth of the yeast *tps1Δ* mutant, and *PvTpp* rescued the growth of the *tps2Δ* mutant, providing evidence that *PvTpsα* and *PvTpp* encode genetically functional trehalose synthases (Fig. 5). Furthermore, we confirmed the enzymatic activities for PvTPSα*in vivo* (Fig. 4E) and PvTPP *in vitro* (Fig. 4B), but not for PvTPSβ. Thus far, all cloned insect *Tps* genes encode both GT-20 and TrePP motifs, and insect TPP has been proposed to be identical to TPS [21–23]. Although *PvTpsα/β* also has both of these motifs, we cloned a *PvTpp* gene distinguishable from *PvTpsα/β* and demonstrated the TPP activity of PvTPP. This is the first report of an insect *Tpp* gene. BlastP and Pfam searches have shown that TPP orthologs possessing only the TrePP motif are likely to occur in several insects, including four dipteran species, such as *Culex quinquefasciatus*, *Anopheles gambiae*, *Aedes aegypti*, *Drosophila melanogaster* and *Drosophila pseudoobscura*, and a hemipteran species, *Maconellicoccus hirsutus* (CPIJ009402 in *C. quinquefasciatus*; AGAP008225 in *Anopheles gambiae*; AAEL010684 in *Aedes aegypti*; CG5171 and CG5177 in *D. melanogaster*; GA18712 and GA18709 in *D. pseudoobscura*; and ABN12077 in *M. hirsutus*). We therefore propose that insect *Tps* and *Tpp* genes exist independently, as reported in other organisms, e.g. bacteria, yeast and plants [32].

**Fig. 7:**
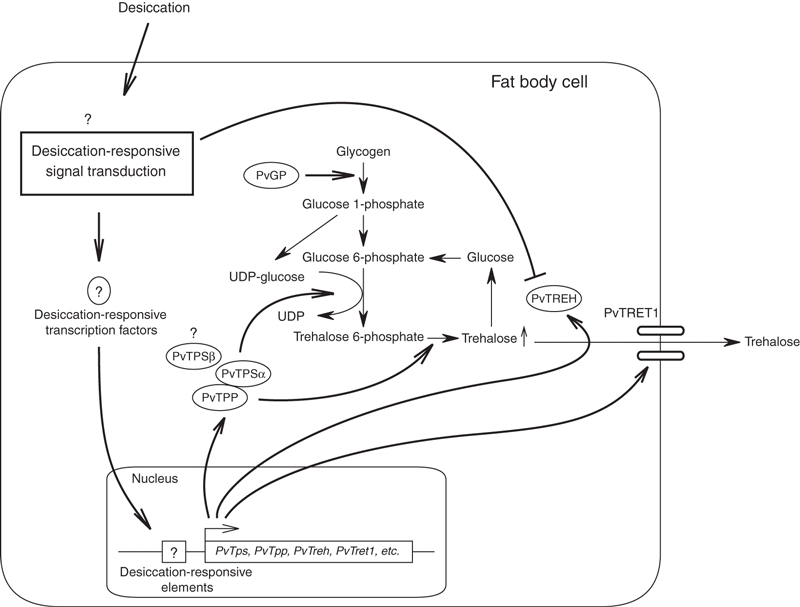
Proposed molecular mechanism of desiccation-inducible trehalose accumulation in *P. vanderplanki*.

In *Saccharomyces cerevisiae*, trehalose synthase forms a heterotetramer with TPS1, TPS2, TPS3 and TSL1 subunits [42,43]. In the complex, the TPS3 and TSL1 subunits, both of which possess GT-20 and TrePP motifs without TPS or TPP activity, act as regulators [27,28,42–44]. In addition, the activity of TPS is enhanced by its aggregation, indicating that heteromeric and/or homomeric multimerization of the TPS–TPP complex should be important for the production of TPS activity [45]. Similar to *S. cerevisiae*, other regulatory subunits might constitute the trehalose synthase complex in *P. vanderplanki*. No cDNAs homologous to TPS3 and TSL1 have been found thus far in the EST database of *P. vanderplanki*. Although we could not detect TPS activity in PvTPSβ (Fig. 5A), acceleration of its expression by desiccation (Fig. 7) suggests that the protein also plays a role in anhydrobiosis induction. PvTPSβ might act as a regulatory subunit, in a similar manner to TPS3 and TSL1, interacting with PvTPSα and PvTPP. The absence of enzymatic activity in PvTPSα/β proteins prepared by an *in vitro* transcription and translation system might be caused by the inappropriate interaction of components. If PvTPSα also possesses the same property as TPS in yeast, aggregation of PvTPSα caused by dehydration could lead to an enhancement of its activity en route to anhydrobiosis. Further investigation is required to answer these questions.

During the induction of dehydration in an anhydrobiotic nematode, *A. avenae*, lipid is used as the most likely carbon source to synthesize trehalose via the glyoxylate cycle, and glycogen degradation also contributes to trehalose synthesis [39,46]. In addition, in the trehalose synthesis mechanism of *A. avenae* during anhydrobiosis induction, it has been reported that the excess substrate influx into TPS is caused by the saturation of glycogen synthase as a result of the increase in UDP-glucose and glucose-6-phosphate as dehydration progresses [47]. However, as shown in Fig. 1B, glycogen degradation and trehalose accumulation during the induction of anhydrobiosis in *P. vanderplanki* occur as a mirror image. This result indicates that, in drying *P. vanderplanki* larvae, glycogen is the largest source of trehalose synthesis and is gradually converted into trehalose to act as an anhydroprotectant, although we have not yet verified the involvement of the glyoxylate cycle. Neither the expression of *PvGp* mRNA nor the activity of GP was elevated on desiccation (Figs 2A and 6A), indicating that PvGP is not involved in the degradation of glycogen. However, TPS and TPP activities increased prior to and parallel with trehalose accumulation, respectively, as a result of the upregulation of the expression of the corresponding mRNAs and proteins (Figs 2B, C and 6A, B). In contrast with the case of TPS and TPP, TREH activity was depressed during desiccation treatment, even though the mRNA and protein of *PvTreh* increased (Figs 2D and 6). These interesting results indicate that trehalose accumulation can be attributed to the enhancement of *PvTps* and *PvTpp* gene expression and the repression of enzymatic activity for PvTREH.

*In vitro* recombinant PvTREH without modification, such as phosphorylation, showed hydrolytic activity (Fig. 4C, D), implying that PvTREH activity in desiccating larvae might be negatively modified post-translationally. In insects, TREH activity is thought to depend on transcriptional regulation, as reported in the ovary and midgut of *B. mori* [48,49], or on the coexistence of a TREH inhibitor, as in the hemolymph of *Periplaneta americana* [50]. In *S. cerevisiae*, TREH is activated through phosphorylation by cdc28 and inactivated by an inhibitor of TREH (DCS1/YLR270W) [51–53]. Post-translational modification of PvTREH activity could be occurring in a similar manner, such as by phosphorylation or the coexistence of an inhibitor for rapid accumulation and breakdown (see [54]) of trehalose, in dehydrated and rehydrated larvae, respectively.

In *P. vanderplanki*, the expression and activity of the enzymes of trehalose metabolism are regulated by desiccation stress (Figs 2 and 6). This is the first report concerning the comprehensive analyses of trehalose metabolism enzymes and the corresponding genes in a single insect species, and provides evidence that multiple pathways control trehalose concentration appropriately according to its physiological role. In insects, including *P. vanderplanki*, trehalose production and utilization as a hemolymph sugar are under hormonal control via the central nervous system under normal conditions [12]. However, in dehydrating *P. vanderplanki* larvae, trehalose accumulation as an anhydroprotectant is independent of the control of the central nervous system [40], and is instead triggered by an increase in internal ion concentration [41]. A requirement for rapid adaptation to a desiccating environment could have led to the evolution of the cell autonomous responsive systems in *P. vanderplanki* larvae.

Here, we summarize a probable molecular mechanism underlying trehalose metabolism that is involved in anhydrobiosis induction in *P. vanderplanki* (Fig. 7). Once larvae are exposed to drying conditions, fat body cells receive the desiccation signal through the elevation of internal ion concentration and rapidly activate certain desiccation-responsive transcription factors to enhance the transcription of *PvTpsα/β* and *PvTpp* genes participating in trehalose synthesis. Indeed, mRNAs of *PvGp*, *PvTpsα/β* and *PvTpp* are abundantly expressed in fat body tissue, but the *PvTreh* mRNA level is less than that in other tissues (Fig. S1, Table S2 and Doc. S1). Furthermore, the PvTPSα/β protein localizes only to fat body tissue (Fig. S2 and Doc. S1). Concomitant with the accumulation of PvTPSα/β and PvTPP proteins, the aggregation of PvTPSα/β–TPP complexes, facilitated by dehydration of the cells, might potentiate the activity of the complex, resulting in the very rapid production of trehalose. Synthesized trehalose then diffuses via the hemolymph through TRET1 to protect all cells and tissues from irreversible desiccation damage (see [7–9]). Just before the completion of anhydrobiosis, the expression of *PvTreh* is accelerated, and the activity of PvTREH is depressed, for subsequent activation during rehydration. Consequently, strict temporal regulation of the pathway of trehalose metabolism, in response to desiccation stress, seems to be the key for the completion of anhydrobiosis in *P. vanderplanki*. Interestingly, *P. nubifer*, a desiccation-sensitive and congeneric chironomid to *P. vanderplanki*, contains trehalose at a comparable level to that in *P. vanderplanki* under normal conditions, but it does not accumulate trehalose during desiccation (data not shown). Therefore, among the chironomid species, *P. vanderplanki* seems to be specifically adapted to dehydration by controlling the expression of trehalose metabolism-related genes and the activities of the proteins. In future studies, the determination of the *cis*-elements and *trans*-factors of *PvTps* and other desiccation-inducible genes will be essential in order to obtain a comprehensive understanding of the regulatory mechanisms underlying the induction of anhydrobiosis. Such an understanding could also lead to the exploitation of desiccation-responsive heterologous gene expression systems that are crucial for the reconstitution of the anhydrobiotic state.

## Experimental procedures

### Insects

*Polypedilum vanderplanki* larvae were reared on a milk agar diet under a controlled photoperiod (13 h light : 11 h dark) at 27 °C [40,55]. Procedures for the desiccation treatment for the induction of anhydrobiosis-related genes have been described previously [41].

### Determination of glycogen and trehalose content in *P. vanderplanki*

Larvae of *P. vanderplanki* desiccated for various periods were homogenized in 80% ethanol to obtain soluble and insoluble fractions. The soluble fractions were prepared for the determination of trehalose as described previously [40]. The insoluble fractions were boiled for 30 min in the presence of 30% KOH; glycogen was then precipitated in 80% ethanol and collected by centrifugation at 20 000 ***g*** for 15 min at room temperature. The resulting glycogen precipitates were dissolved in distilled water. The glycogen content was determined by the phenol–sulfuric acid method [56].

### Cloning of *PvTps*, *PvTpp*, *PvTreh* and *PvGp* cDNAs

Full-length cDNAs of *PvTps*, *PvTreh* and *PvGp* were isolated by RT-PCR with degenerated primers and/or by 5′- and 3′-RACE with a SMART RACE cDNA amplification kit (Clontech, Mountain View, CA). The degenerated primers used for RT-PCR were as follows: PvTPS-F1, 5′-GACTCITAYTAYAAYGGITGYTGYAA-3′; PvTPS-F2, 5′-TGGCCIYTITTYCAWSIATGCC-3′; PvTPS-R1, 5′-GGRAAIGGIATWGGIARRAARAA-3′; PvTPS-R2, 5′-ARCATIARRTGIACRTCWGG-3′; PvTREH-F1, 5′-ATHRTICCIGGIGGIMGITT-3′; PvTREH-R1, 5′-TTIGGIDMRTCCCAYTGYTC-3′; PvGP-F1, 5′-AAYGGIGGIYTIGGIMGIYTIGCIGC-3′; PvGP-R1, 5′-TGYTTIARICKIARYTCYTTICC-3′. *PvTpp* cDNA was obtained from the Pv-EST database [33] and subsequent 5′-RACE. The primers for 5′- and 3′-RACE are shown in Table S1. The nucleotide sequences for the isolated cDNAs were analyzed by GENETYX-MAC (Genetyx, Tokyo, Japan) with the Pv-EST database and subcloned into the appropriate vectors for subsequent experiments. The deduced amino acid sequences of PvTPSα/β, PvTPP and PvTREH were subjected to Pfam search (pfam.sanger.ac.uk) for motif analysis.

### Determination of the *PvTps* gene structure

Genomic DNA was extracted from the larvae of *P. vanderplanki* using a DNeasy Tissue Kit (Qiagen, Hilden, Germany). The construction of the fosmid library and the screening of the clones containing the *PvTps* gene were entrusted to TaKaRa Bio Inc., Shiga, Japan. The positive clones were subjected to sequencing analysis, and the structure of the *PvTps* gene was determined. The primer sets used are shown in Table S1.

### Northern blot analysis

Total RNA was isolated from dehydrating larvae using TRIzol (Invitrogen, Carlsbad, CA). Northern blot analysis was performed as described previously [9,33]. Briefly, 15 μg of RNA was electrophoresed on 1% agarose–20 mm guanidine isothiocyanate gels, blotted onto Hybond N-plus membrane (GE Healthcare Bioscience, Piscataway, NJ) and probed with the full length of the corresponding cDNA fragments labeled with [α-^32^P]dATP using a Strip-EZ labeling kit (Ambion, Austin, TX). The hybridized blot was analyzed by BAS 2500 (Fuji Film, Tokyo, Japan).

### Protein extraction

For western blot analyses, the larvae were homogenized in a 10-fold volume of SDS/PAGE sample buffer without dye reagent, and boiled for 10 min. The homogenates were centrifuged at 20 000 ***g*** for 10 min at room temperature, and the supernatants were collected. The concentration of protein was determined as described previously [14]. The preparation of yeast protein extract was carried out according to Clontech’s Yeast Protocols Handbook (PT3024-1; http://www.clontech.com). For the determination of enzyme activities, the larvae were homogenized in a 20-fold volume of protein extraction buffer (T-PER; Pierce Biotechnology, Rockford, IL) containing a protease inhibitor cocktail (Complete; Roche Diagnostics, Basel, Switzerland), and the supernatants containing the crude protein were obtained by centrifugation at 20 000 ***g*** for 5 min at 4 °C. The concentration of protein was determined with a BCA Protein Assay Kit (Bio-Rad, Hercules, CA).

### Western blot analysis

Using the protein extracts described above, western blot analysis was performed as described previously [9,33]. The blots were treated with anti-PvTPS, TPP or TREH polyclonal IgGs as the primary antibodies to detect the corresponding proteins, and subsequently with goat anti-rabbit IgG (H + L) conjugated with horseradish peroxidase (American Qualex, La Mirada, CA) as the secondary antibody, and reacted with Immobilon Western Chemiluminescent HRP substrate (Millipore, Billerica, CA) to analyze the chemiluminescent signals by LAS-3000 (Fuji Film). The recognition sites of antibodies for PvTPS, TPP and TREH are the following amino acid sequences: (592)GIEGITYAGNHGLE(605) of PvTPSα/β, (108)GIDGIVYAGNHGLE(121) of PvTPP and (109)LDKISDKNFRD(119) of PvTREH.

### *In vitro* transcription and translation

*In vitro* transcription and translation of PvTPSα/β, PvTPP and PvTREH were performed using a TnT® T7 Quick for PCR DNA kit (Promega). Briefly, approximately 200 ng of each PCR product, flanked by a T7 promoter at the 5′-end and a poly(A) at the 3′-end of the ORF, were incubated for 90 min at 30 °C in a 50-μL reaction mixture containing 1 μL of 1 mm methionine or [^35^S]methionine (> 37 TBq·mmol^−1^, 400 MBq·mL^−1^; Muromachi Chemical, Tokyo, Japan). The reaction products were separated by 15% SDS/PAGE, and the gel was applied to western blot analyses as described above, or for autoradiography to confirm protein synthesis.

### Determination of enzyme activity

GP (EC 2.4.1.1) assays were performed as follows: 100 μL of 45 mm potassium-phosphate buffer (pH 6.8), containing 0.1 mm EDTA, 15 mm MgCl_2_, 4 μm glucose-1,6-bisphosphate, 0.1 U phosphoglucomutase, 0.6 U glucose-6-phosphate dehydrogenase, 2 mg·mL^−1^ glycogen, 0.4 mm NADP and 10 μL of protein extract, were incubated at 30 °C for 30 min, monitoring the change in the absorbance at 340 nm (*A*_340_). Because the inactive form of GP is activated by an allosteric effector, such as AMP, to determine total GP (active ‘a’ form and inactive ‘b’ form) activity, the reactions were performed in the presence of an additional 1 mm 5′-AMP.

For TPS assays, 200 μL of reaction mixture, containing 2.5 mm glucose-6-phosphate, 2.5 mm UDP-glucose, 2.5 mm MgCl_2_, 100 mm KCl, 1.25 mm phosphoenolpyruvate, 20 μL pyruvate kinase/lactate dehydrogenase (34 μL·mL^−1^), 0.3 mm NADH, 30 mm Tris/HCl (pH 7.4) and 5 μL of protein extract, were incubated at 30 °C for 30 min, monitoring the change in *A*_340_ that depends on NADH oxidation. In the case of samples from *in vitro* transcription and translation, 1.2 μL each of the products were incubated at 30 °C for 2 h, and then at 95 °C for 10 min to stop the reaction.

Assays for TPP activity were performed in 200 μL of reaction mixture containing 2.5 mm trehalose-6-phosphate, 2.5 mm MgCl_2_, 30 mm Tris/HCl (pH 7.4) and 20 μL of protein extract. In assays for the *in vitro* transcription and translation products, 1.2 μL of each of the preparations was used. The mixtures were incubated at 30 °C for 1 h, and then at 95 °C for 10 min to stop the reaction. The reaction product (trehalose) was measured by HPLC [40].

TREH activity was assayed in 250 μL of 15 mm phosphate buffer (pH 6.0) containing 20 mm trehalose and an appropriate amount of protein preparation. After incubation at 30 °C for 0.5–1 h, the reaction mixture was boiled for 5 min. As a control, another reaction mixture was immediately boiled without incubation. The reaction products (trehalose and glucose) were measured by HPLC [40].

A desiccation treatment of 48 h is required to completely desiccate larvae under laboratory conditions [40,41]. Enzyme activities in the larvae were measured from 0 to 40 h after the beginning of desiccation, as it seems likely that no metabolic activity would be detectable *in vivo* in completely desiccated larvae [2].

### Yeast complementation assay

The *S. cerevisiae* deletion mutants were purchased from Open Biosystems, Huntsville, AL. The deletion strains for *TPS1* (*MATα*; *his3Δ1*; *leu2Δ0*; *lys2Δ0*; *ura3Δ0*; *YBR126c::kanMX4*) and *TPS2* (*MATα*; *his3Δ1*; *leu2Δ0*; *lys2Δ0*; *ura3Δ0*; *YDR074w::kanMX4*) were transformed with pUG35 (http://mips.gsf.de/proj/yeast/info/tools/hegemann/gfp.html; U. Gueldener and J. H. Hegemann, Heinrich-Heine-Universität Düsseldorf, unpublished results), which contains the ORF of *PvTpsα*, *PvTpsβ* and *PvTpp* under the MET25 promoter [57]. For the positive and negative controls, wild-type and deletion mutants were transformed with pUG35 containing the *GFP* ORF instead of the target genes. After selection on synthetic defined (SD) medium lacking uracil, transformants were confirmed by colony PCR. Three independent colonies were picked up for each strain. For the complementation test of the *tps1* mutant, transformants of the *tps1* deletion mutant with *PvTpsα* and *PvTpsβ* were grown in SD medium containing 2% galactose and lacking uracil and methionine at 30 °C to an exponential phase. After harvesting of the yeast cells, a dilution series of 10^4^–10^1^ cells was prepared, and each solution was spotted onto yeast extract and peptone (YP) medium containing galactose conditioned in hyperosmolarity with 1 m NaCl, 50% sucrose or 1.5 m sorbitol. For complementation tests of the *tps2* mutant, diluted series of transformants of the *tps2* deletion mutant with *PvTpp* were prepared as for *tps1*. Each cell suspension was spotted onto SD medium containing 2% galactose and lacking methionine and uracil. To confirm the rescue of the temperature sensitivity of the *tps2Δ* mutant, the plates were incubated at 45 °C for 5 h and then at 30 °C for 3–4 days.

### Quantification of trehalose by HPLC

The amount of trehalose was determined by HPLC according to Watanabe *et al.* [40]. For the determination of intracellular trehalose content, *PvTpsα*- or *PvTpsβ*-introduced yeast strains were cultured in SD medium containing galactose and lacking uracil and methionine at 30 °C for 48 h until the growth curve entered the stationary phase. Yeast cells were harvested and homogenized with glass beads in 80% ethanol. After centrifugation at 20 000 ***g*** for 30 min, the supernatants were collected and subjected to sample preparation for HPLC analysis [40].

## Abbreviations

EST: expressed sequence tag
GP: glycogen phosphorylase
GT-20: glycosyl transferase family 20
TPP: trehalose-6-phosphate phosphatase
TPS: trehalose-6-phosphate synthase
TREH: trehalase
TrePP: trehalose-phosphatase

## Supporting information

The following supplementary material is available:

**Fig. S1**. Tissue specificity of expression of *PvGp, PvTps* α/β, *PvTpp* and *PvTreh* in *P. vanderplanki* larvae.

**Fig. S2**. Immunostaining of PvTPS protein in desiccating larvae.

**Doc S1**. Experimental procedures for supplementary data.

**Table S1**. Primers for 5′- and 3′-RACE, and for the determination of *PvTps* gene structure.

**Table S2**. Primers for real-time PCR.

This supplementary material can be found in the online version of this article.

Please note: As a service to our authors and readers, this journal provides supporting information supplied by the authors. Such materials are peer-reviewed and may be re-organized for online delivery, but are not copy-edited or typeset. Technical support issues arising from supporting information (other than missing files) should be addressed to the authors.
