# Supplementary material for: Enzymatic control of anhydrobiosis-related accumulation of trehalose in the sleeping chironomid, Polypedilum vanderplanki
Source: FEBS J. 2010 Oct;277(20):4215–28. doi: 10.1111/j.1742-4658.2010.07811.x (PMC3037560; doi:10.1111/j.1742-4658.2010.07811.x)
Supplement: Supplementary file 1 [file febs0277-4215-SD1.zip › ejb_7811_sm_Doc S1_TableS1_S2_FigsS1_S2.pdf]

the FEBS Journal
www.febsjournal.org

Enzymatic control of anhydrobiosis-related accumulation of trehalose in the sleeping chironomid, *Polypedilum vanderplanki*

Kanako Mitsumasu, Yasushi Kanamori, Mika Fujita, Ken-ichi Iwata, Daisuke Tanaka, Shingo Kikuta, Masahiko Watanabe, Richard Cornette, Takashi Okuda and Takahiro Kikawada

DOI: 10.1111/j.1742-4658.2010.07811.x

EXPERIMENTAL PROCEDURES FOR SUPPEMENTARY DATA

Quantitative PCR

Total RNA was isolated from various tissues in *P. vanderplanki* under normal conditions (fully hydrated) using RNAiso Plus reagent (TaKaRa Bio, Shiga, Japan). The cDNA was synthesized from 400 ng of the total RNA using the PrimeScript RT reagent kit (Takara Bio). PCR amplification was performed with the CFX96 Real-Rime PCR Detection System (Bio-Rad, Hercules, CA) with SYBR Premix Ex Taq (TaKaRa Bio). PCR conditions were 95˚C for 10 s followed by 40 cycles of 95˚C for 5 s each, 60˚C for 10 s each and 72˚C for 10 s each. Primer sets are described in Table S2. Data were analyzed with Bio-Rad CFX Manager version 1.1. Results were normalized by delta-delta Ct analysis with expression levels of reference genes, ribosomal protein L32 and elongation factor 1 alpha (*PvRpl32*, DDBJ accession no. AB244986; and *PvEf1α*, DDBJ accession no. AB490338).

Immunohistochemical analysis

Desiccating larvae of *P. vanderplanki* were placed in cold fixative (1.4 g/100ml of picric acid, formaldehyde and acetic acid in phosphate buffered saline, PBS) for 16 h at 5˚C, dehydrated through a graded series of alcohols to 100% EtOH and xylene, each for 30 min, and then embedded in paraffin (Histosec pastilles, Merck KGaA, Damstadt, Germany). Paraffin blocks were cut into long thin strips which were then mounted on stubbed wooden blocks. Semi-thin sections of 5 μm were de-paraffinized in xylene and rehydrated thorough a graded series of alcohols to PBS at room temperature (RT). After blocking with 10% goat serum (S-1000, Vector Laboratories, Inc., CA, USA) in PBS overnight at 5˚C, these sections were hybridized with each rabbit anti-PvTPS polyclonal antibody (at 1:500 dilution) overnight at 5˚C.

The sections were developed using ABC (avidin-biotin-peroxidase detection system) Vectorstain Elite kit following the manufacture's instruction, and sections were stained with 3,3'-diaminobenzidine (DAB, Vector Laboratories, Inc., city, CA) solution and counterstained in Mayer's hematoxylin at RT and washed in distilled water. The sections were then mounted with Entellan New (Merck KGaA, Damstadt, Germany) after being dehydrated through a graded series of alcohols, and cleared in xylene. Sections were observed by light microscopy (Axio imager A1, Carl Zeiss, Berlin, Germany). Images were photographed using Axio Cam (MRc, Carl Zeiss, Berlin, Germany).

Table S1. Primers for 5'- and 3'-RACE, and for the determination of *PvTps* gene structure.

Purpose	Primer name	Sequence
PvTps cloning (RACE)	PvTPS3'RACE-F1	TGCTGCAATGGAACACATTG
PvTps cloning (RACE)	PvTPS3'RACE-F2	CTTTTCCATTCAATGCCG
PvTps cloning (RACE)	PvTPS3'RACE-F3	GACTCGTATTATAACGGGTGCTGCAATGGAACAC
PvTps cloning (RACE)	PvTPS3'RACE-F4	CTGCAATGGAACACATTGGCCTCTTTTCCA
PvTps cloning (RACE)	PvTPS3'RACE-F5	GGTCGTGCTATTTTCTCAACTGATCACTGGAGAGCGTATG
PvTps cloning (RACE)	PvTPS3'RACE-F6	GCCGCTAACATTATGTTTGCTGATAAGACCATTGAAGCAATTG
PvTps cloning (RACE)	PvTPS3'RACE-F7	GTCGTGTTGATCGCAAAAATCTTTTAGTTGAACATGGTGG
PvTps cloning (RACE)	PvTPS3'RACE-F8	TTCCTATCCATATGTCATTCGACTTAACCACCTCCCCTTC
PvTps cloning (RACE)	PvTPS5'RACE-R1	GCAAGTTGAATGTTGAGATTCTTTTCTTCAGC
PvTps cloning (RACE)	PvTPS5'RACE-R2	TTTGCACAAAGCATCAAATGATAATCATG
PvTps cloning (RACE)	PvTPS5'RACE-R3	CAATTGCTTCAATGGTCTTATCAGCAAACATAATGTTAGCGGC
PvTps cloning (RACE)	PvTPS5'RACE-R4	CATACGCTCTCCAGTGATCAGTTGAGAAAATAGCACGACC
PvTps cloning (RACE)	PvTPS5'RACE-R5	GTTTGATCGTTTGGATCAGATTCGGGAATTTGTAAGTC
PvTpp cloning (RACE)	PvTPP5'RACE-R1	GTCTTCACATCATCAACTCCCCTTCCGGA
PvTpp cloning (RACE)	PvTPP5'RACE-R2	TGGCAGCCAACGTGCCGTCA
PvTreh cloning (RACE)	PvTREH3'RACE-F1	TATATTATTCGTTACGTTCTCAGCCGCCACTTCTTGCACC
PvTreh cloning (RACE)	PvTREH3'RACE-F2	GGCAAATCACACAGTACAAGTTAAAGGACACACATTAGCA
PvTreh cloning (RACE)	PvTREH3'RACE-F3	TGGGATTGGCCAAATGTATGGGCACCAACGCA
PvTreh cloning (RACE)	PvTREH3'RACE-F4	ACTCGGCGGACATGGTGGAGGTGGTGA
PvTreh cloning (RACE)	PvTREH5'RACE-R1	TGCTAATGTGTGTCCTTTAACTTGTACTGTGTGATTTGCC
PvTreh cloning (RACE)	PvTREH5'RACE-R2	GGTGCAAGAAGTGGCGGCTGAGAACGTAACGAATAATATA
PvGp cloning (RACE)	PvGP3'RACE-F1	GTTGTCCATGGGAAAAAGCTCGTCCTGAATACATGATTCC
PvGp cloning (RACE)	PvGP3'RACE-F2	AGTCAACTTTTTTGGACGTGTCATGGACACTCCTGATGGA
PvGp cloning (RACE)	PvGP3'RACE-F3	GGGATCGTATGCGCCGCATGTCA
PvGp cloning (RACE)	PvGP3'RACE-F4	TGCTGTCAACGGTGTCGCTGCA
PvGp cloning (RACE)	PvGP5'RACE-R1	TCCATCAGGAGTGTCCATGACACGTCCAAAAAAGTTGACT
PvGp cloning (RACE)	PvGP5'RACE-R2	GGAATCATGTATTCAGGACGAGCTTTTTCCCATGGACAAC
PvTps gene structure determination	PvTPSGENE-F1	TGACAAAGGAAAGTGATCCAAAACA
PvTps gene structure determination	PvTPSGENE-F2	CTTTCATTGATTATTTTAAACGATGTG
PvTps gene structure determination	PvTPSGENE-F3	AATTTCATCAATCGATCTATCACAC
PvTps gene structure determination	PvTPSGENE-F4	CAACCGCTGGTTTAAAATCAGAG
PvTps gene structure determination	PvTPSGENE-F5	GGAATGCTTGCTTGCGATATG
PvTps gene structure determination	PvTPSGENE-F6	CAGCAGTTGCTCTTGTGACACC
PvTps gene structure determination	PvTPSGENE-F7	CTACGCTTCCACAAGAAACAAAG
PvTps gene structure determination	PvTPSGENE-F8	CATTTGGTGTTGATTGGAGTG
PvTps gene structure determination	PvTPSGENE-F9	TAATCTTGATAGGTTCAATAG
PvTps gene structure determination	PvTPSGENE-R1	TTCCAACCATGTTTTTAACATTTTCAAC
PvTps gene structure determination	PvTPSGENE-R2	AATTTGTTCGAATTTACCATCATTGCG

Table S2. Primers for real time-PCR.

Gene	Forward primer	Reverse primer
PvEfα1	AACTGACAAACCATTGCG	TCACCTGGTACAGCTTCT
PvRpl32	AACTTAAGAGGAATTGGCGT	ATTTCCAGCTCTTTAACATTGTG
PvGp	TTAAAAAGCAAGACGAAGTCTCTGC	TATGAAGGCTGAACACCCCAA
PvTps	GAATGAGCAGATTACGTCGTAG	CTTGTGTGAATAGCCGATGTA
PvTpp	TATCCTAATGGAACTCGTTACATACAC	CTTGCCCTTATGCCATACT
PvTreh	AACTTATGTTGAAACCACAAATGA	TTCTGCATAATGTTCTTCTTTGTCT

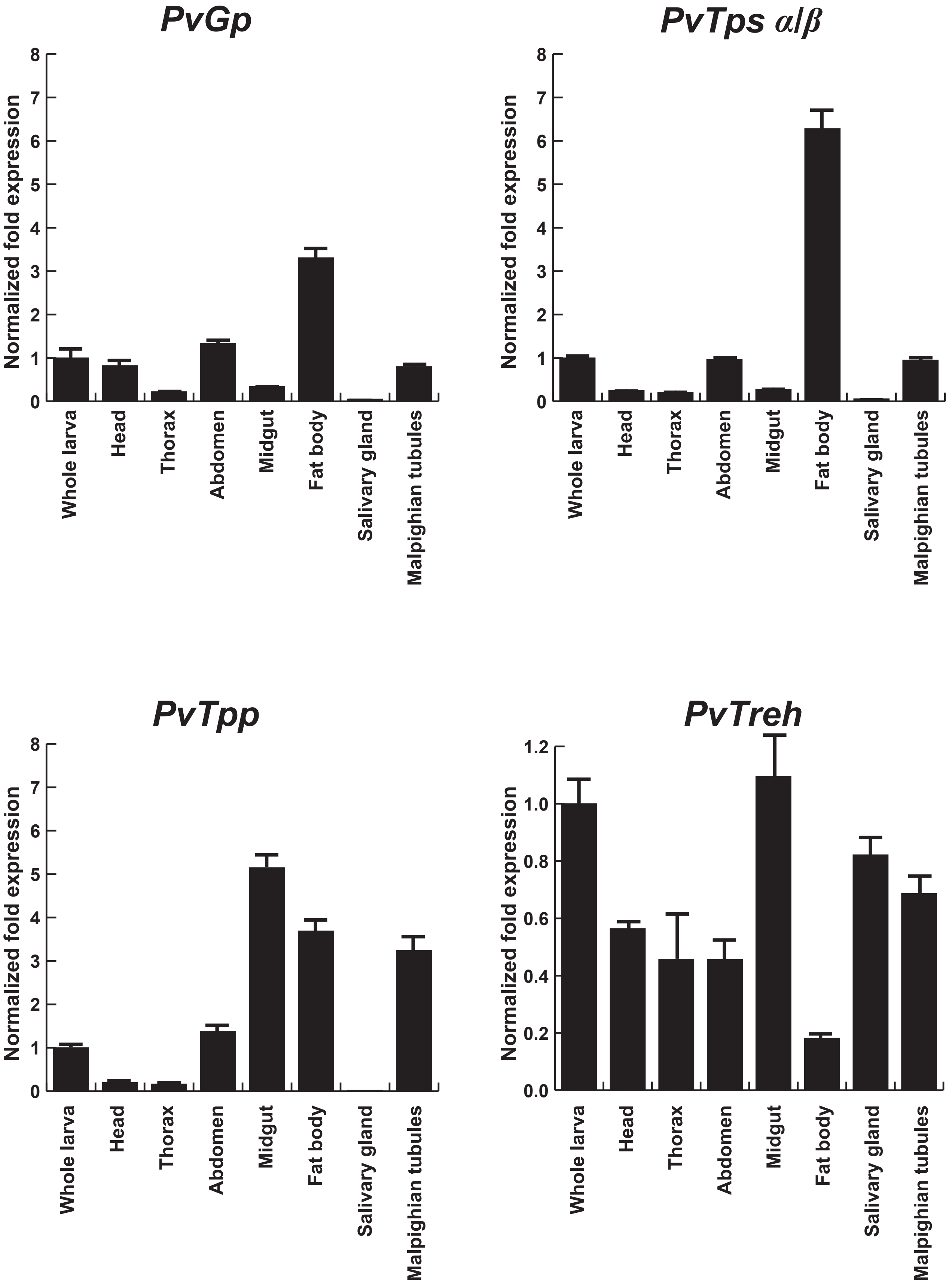

Figure S1.

Tissue-specificity of expression of *PvGp, PvTps α/β, PvTpp* and *PvTreh* in *P. vanderplanki* larvae. Expression levels of the corresponding genes were measured by quantitative PCR with cDNA obtained from various tissues in larvae. The relative quantities of the normalized expression levels for the corresponding genes in the whole larvae are indicated as 1.

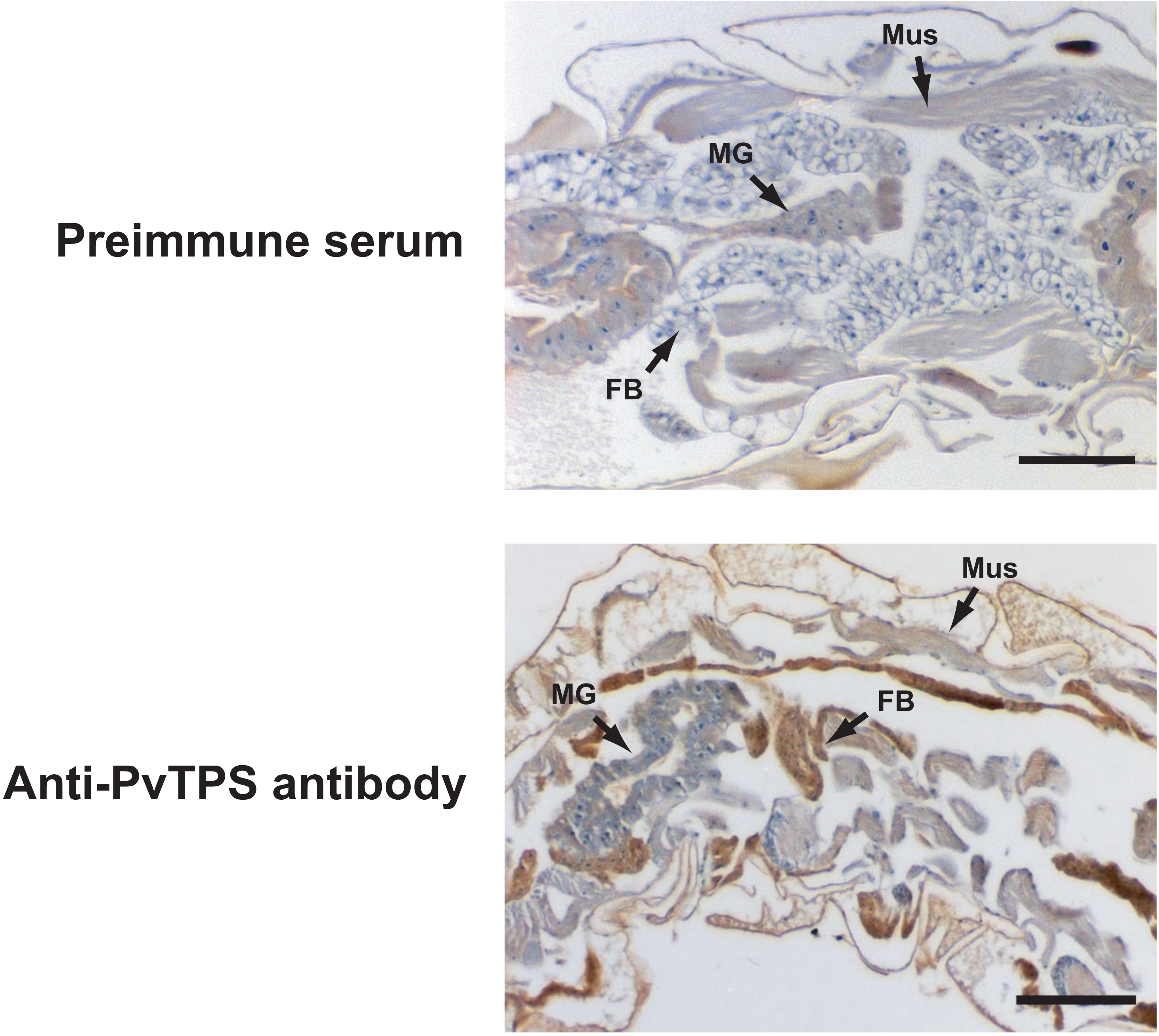

Figure S2.

Immunostaining of PvTPS protein in desiccating larvae. Upper panel, sagittal section of larvae stained with pre-immune serum as a control; Lower panel, staining with anti-PvTPS polyclonal antibody. Positive staining was observed only in fat body. FB, fat body; MG, midgut; Mus, muscle. Scale bar, 100μm.
